# Digital auscultation in PERCH: Associations with chest radiography and pneumonia mortality in children

**DOI:** 10.1002/ppul.25046

**Published:** 2020-09-11

**Authors:** Eric D. McCollum, Daniel E. Park, Nora L. Watson, Nicholas S. S. Fancourt, Christopher Focht, Henry C. Baggett, W. Abdullah Brooks, Stephen R. C. Howie, Karen L. Kotloff, Orin S. Levine, Shabir A. Madhi, David R. Murdoch, J. Anthony G. Scott, Donald M. Thea, Juliet O. Awori, James Chipeta, Somchai Chuananon, Andrea N. DeLuca, Amanda J. Driscoll, Bernard E. Ebruke, Mounya Elhilali, Dimitra Emmanouilidou, Louis Peter Githua, Melissa M. Higdon, Lokman Hossain, Yasmin Jahan, Ruth A. Karron, Joshua Kyalo, David P. Moore, Justin M. Mulindwa, Sathapana Naorat, Christine Prosperi, Charl Verwey, James E. West, Maria Deloria Knoll, Katherine L. O'Brien, Daniel R. Feikin, Laura L. Hammitt

**Affiliations:** ^1^ Global Program in Respiratory Sciences, Eudowood Division of Pediatric Respiratory Sciences Johns Hopkins School of Medicine Baltimore Maryland USA; ^2^ Department of International Health Johns Hopkins Bloomberg School of Public Health Baltimore Maryland USA; ^3^ Department of International Health, International Vaccine Access Center Johns Hopkins Bloomberg School of Public Health Baltimore Maryland USA; ^4^ Department of Epidemiology and Biostatistics, Milken Institute School of Public Health George Washington University Washington District of Columbia USA; ^5^ The Emmes Corporation Rockville Maryland USA; ^6^ Global Disease Detection Center, US Centers for Disease Control and Prevention Collaboration Thailand Ministry of Public Health Mueang Nonthaburi Nonthaburi Thailand; ^7^ Division of Global Health Protection, Center for Global Health Centers for Disease Control and Prevention Atlanta Georgia USA; ^8^ International Centre for Diarrhoeal Disease Research, Bangladesh (icddr,b) Dhaka and Matlab Bangladesh; ^9^ Medical Research Council Unit Basse The Gambia; ^10^ Department of Paediatrics University of Auckland Auckland New Zealand; ^11^ Centre for International Health University of Otago Dunedin New Zealand; ^12^ Division of Infectious Disease and Tropical Pediatrics, Department of Pediatrics, Center for Vaccine Development and Global Health University of Maryland School of Medicine Baltimore Maryland; ^13^ Bill & Melinda Gates Foundation Seattle Washington USA; ^14^ Medical Research Council: Respiratory and Meningeal Pathogens Research Unit University of the Witwatersrand Johannesburg South Africa; ^15^ Department of Science and Technology/National Research Foundation: Vaccine Preventable Diseases Unite University of the Witwatersrand Johannesburg South Africa; ^16^ Department of Pathology and Biomedical Science University of Otago Christchurch New Zealand; ^17^ Microbiology Unit Canterbury Health Laboratories Christchurch New Zealand; ^18^ Kenya Medical Research Institute‐Wellcome Trust Research Programme Kilifi Kenya; ^19^ Department of Infectious Disease Epidemiology London School of Hygiene & Tropical Medicine London UK; ^20^ Department of Global Health Boston University School of Public Health Boston Massachusetts USA; ^21^ Department of Paediatrics and Child Health University Teaching Hospital Lusaka Zambia; ^22^ Department of Epidemiology Johns Hopkins Bloomberg School of Public Health Baltimore Maryland USA; ^23^ International Foundation Against Infectious Disease in Nigeria Abuja Nigeria; ^24^ Department of Electrical and Computer Engineering Johns Hopkins University Baltimore Maryland USA; ^25^ Department of International Health, Center for Immunization Research Johns Hopkins Bloomberg School of Public Health Baltimore Maryland USA; ^26^ Department of Paediatrics, Faculty of Health Sciences University of the Witwatersrand Johannesburg South Africa

**Keywords:** child, developing countries, digital auscultation, radiography, respiratory tract infections

## Abstract

**Background:**

Whether digitally recorded lung sounds are associated with radiographic pneumonia or clinical outcomes among children in low‐income and middle‐income countries is unknown. We sought to address these knowledge gaps.

**Methods:**

We enrolled 1 to 59monthold children hospitalized with pneumonia at eight African and Asian Pneumonia Etiology Research for Child Health sites in six countries, recorded digital stethoscope lung sounds, obtained chest radiographs, and collected clinical outcomes. Recordings were processed and classified into binary categories positive or negative for adventitial lung sounds. Listening and reading panels classified recordings and radiographs. Recording classification associations with chest radiographs with World Health Organization (WHO)‐defined primary endpoint pneumonia (radiographic pneumonia) or mortality were evaluated. We also examined case fatality among risk strata.

**Results:**

Among children without WHO danger signs, wheezing (without crackles) had a lower adjusted odds ratio (aOR) for radiographic pneumonia (0.35, 95% confidence interval (CI): 0.15, 0.82), compared to children with normal recordings. Neither crackle only (no wheeze) (aOR: 2.13, 95% CI: 0.91, 4.96) or any wheeze (with or without crackle) (aOR: 0.63, 95% CI: 0.34, 1.15) were associated with radiographic pneumonia. Among children with WHO danger signs no lung recording classification was independently associated with radiographic pneumonia, although trends toward greater odds of radiographic pneumonia were observed among children classified with crackle only (no wheeze) or any wheeze (with or without crackle). Among children without WHO danger signs, those with recorded wheezing had a lower case fatality than those without wheezing (3.8% vs. 9.1%, *p* = .03).

**Conclusions:**

Among lower risk children without WHO danger signs digitally recorded wheezing is associated with a lower odds for radiographic pneumonia and with lower mortality. Although further research is needed, these data indicate that with further development digital auscultation may eventually contribute to child pneumonia care.

## 1 INTRODUCTION

According to 2017 global estimates, pneumonia is the leading infectious cause of death among children 1–59 months of age annually.^1^ About 80% of these deaths occur in sub‐Saharan Africa and southern Asia.^1^ Child pneumonia management recommendations in sub‐Saharan African and southern Asian countries are commonly based on World Health Organization (WHO) guidelines which are highly sensitive to ensure children likely to have bacterial pneumonia receive antibiotics.^2^, ^3^, ^4^

Recent research from the Pneumonia Etiology Research for Child Health (PERCH) study suggests that the epidemiology of lower respiratory infections among children in developing countries is shifting towards viral causes, a transition likely accelerated by the introduction of *Haemophilus influenzae* type b and pneumococcal conjugate vaccines in these regions.^5^, ^6^ This epidemiologic transition, along with rising rates of antimicrobial resistance, has important implications for application of the WHO guidelines.^7^ Both issues potentially escalate the need for the guidelines to reduce misdiagnosis and antibiotic overtreatment. Ancillary diagnostics that are feasible for low‐income and middle‐income countries (LMICs), are needed.

The acoustic stethoscope is an important diagnostic tool, its origins dating back to the early 1800s.^8^ While many consider chest auscultation with a stethoscope synonymous with medical care, it is not included as a diagnostic in the WHO pneumonia primary care guidelines where most children first access care. This is likely because teaching lung auscultation with acoustic stethoscopes requires medical expertise and time, both of which are not readily available in often overburdened primary care clinics in LMICs. Furthermore, lung sound interpretation with acoustic stethoscopes is largely considered subjective, achieving modest agreement between experienced physicians.^9^, ^10^ Children pose additional challenges given their breathing patterns can vary, as can their cooperation, contributing to breath‐to‐breath lung sound variation.

Digital stethoscopes may overcome these challenges. They are portable electronic devices that can noninvasively transmit, filter, and amplify lung sounds for real‐time human interpretation.^11^ Digital stethoscopes can also record lung sounds to allow post‐processing of sound recordings, more rigorous human interpretation, and computerized automated analysis.^12^, ^13^ In LMICs with limited resources, digitally recorded lung sounds have the potential for use in research and, with further advancements, as a point‐of‐care respiratory diagnostic during clinical care or in the emerging field of telemedicine. Currently little is known whether digitally recorded lung sounds, when interpreted acoustically by humans, are associated with clinical outcomes or radiographic disease among children in LMICs.

During PERCH we used a digital stethoscope to record lung sounds from children 1–59 months of age hospitalized with WHO‐defined clinical pneumonia in six sub‐Saharan African and South Asian countries.^14^ Our objectives for this research were twofold. First, we aimed to evaluate the association of digitally recorded lung sounds with WHO‐defined radiographic primary endpoint pneumonia, and, second, we sought to determine whether digitally recorded lung sounds are associated with mortality among PERCH children with WHO‐defined clinical pneumonia.

## 2 MATERIALS AND METHODS

### 2.1 PERCH enrollment

The PERCH study prospectively enrolled hospital cases and community controls over a 2‐year period at each site in seven countries in Africa and Asia.^5^ As previously described, from December 2012 to January 2014 hospitalized children 1–59 months of age who were eligible for PERCH in Bangladesh, The Gambia, Kenya, South Africa, Thailand, and Zambia could have their lung sounds recorded during enrollment; the Mali site did not participate.^14^ Cases were eligible if 1–59 months old and they met pre‐2013 WHO severe or very severe pneumonia criteria (Table 1). If the child with chest indrawing in the absence of danger signs was found to be wheezing during enrollment screening they received bronchodilator treatment, and if chest indrawing was present and subsequently resolved after treatment they were excluded.^5^ Antero‐posterior chest radiographs were obtained on cases at admission and interpreted by a panel of physicians standardized to interpret chest radiographs per the WHO research methodology.^15^, ^16^ See Table 1 for WHO chest radiograph classifications. Discharge status and hospital outcome were recorded, and children discharged alive were followed up 30 days after hospital admission to obtain vital status. PERCH clinical study staff, which included a mix of nurses, nonphysician clinicians, and physicians, which varied by study site, received intensive clinical training on respiratory assessments and laboratory and radiographic procedures before study commencement and throughout the study at regular frequencies.^17^, ^18^

**Table 1:** Study definitions

**Cases**	
**(Adapted from PERCH et al.)** ^5^	
WHO severe pneumonia	Cough and/or difficult breathing with lower chest indrawing and no WHO danger signs (central cyanosis, difficulty breastfeeding or drinking, vomiting everything, convulsions, lethargy or unconsciousness, head nodding).
WHO very severe pneumonia	Cough and/or difficult breathing with at least one danger sign.
**WHO chest radiographic classifications**	
**(Adapted from Fancourt N et al**.^15^ **and Cherian T et al**.^16^ **)**	
Radiographic pneumonia^a^	An opacity that includes a portion or whole of a lobe, or the entire lung, that is dense or fluffy in appearance and may or may not contain air bronchograms. An opacity of any size or density that creates a silhouette sign with the mediastinal border. An opacity of any size or density associated with a pleural effusion in the lateral pleural space at the costophrenic angle or adjacent lateral chest wall. May not be associated with an opacity if the effusion occludes a majority of the hemithorax (opacity assumed). Pleural effusion does not include fluid in the horizontal or oblique fissures.
Other infiltrate	Densities in both lungs that appear linear, patchy, and lacy (interstitial infiltrate) includes peribronchial thickening and atelectasis; can also be smaller patchy infiltrates or atelectasis that does not meet the criteria of radiographic pneumonia.
Uninterpretable	Image is not interpretable regarding the presence or absence of radiographic pneumonia.
**Digitally recorded lung sound models**	
**(Adapted from McCollum ED et al**.^14^ **)**	
Normal	Soft sounds, not musical or popping in quality.
Crackle only	Short, explosive, not musical, popping sounds; usually repetitive and occurs without wheezes.
Wheeze only	Musical sounds of long duration; can be high or low pitch and occurs without crackles.
Any wheeze	Musical sounds of long duration; can be high or low pitch and can be present with crackles.
Uninterpretable	Persistent crying or poor quality such that no full breath sounds are heard

Abbreviation: WHO, World Health Organization.

a Radiographic pneumonia is termed “primary endpoint pneumonia” in the WHO methodology.

### 2.2 Digital auscultation sampling in PERCH

Sampling of children for lung sound recordings varied by site.^14^ All cases in Matlab and Dhaka, Bangladesh, Sa Kaeo, and Nakhon Phanom, Thailand, and Lusaka, Zambia were consecutively enrolled between September and December 2013, March 2013 and January 2014, and November 2012 and October 2013, respectively. Due to human resource limitations, a convenience sample of cases was enrolled in Kilifi, Kenya between December 2012 and October 2013, in Basse, The Gambia between December 2012 and November 2013, and in Soweto, South Africa between December 2012 and August 2013.

### 2.3 Lung sound recordings

**Figure 1:**
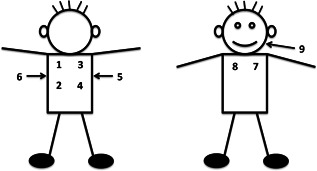
Listening positions for sequential lung sound recordings

All study staff were trained to record lung sounds according to a protocol using a commercial digital stethoscope (ThinkLabs ds32a).^14^ The same study staff that enrolled children into PERCH typically recorded lung sounds on patients. Lung sound recordings were not used to inform clinical care decision‐making. The stethoscope was modified with an external microphone (Sony ECM‐ES30P) that recorded environmental sounds onto a voice recorder (Sony‐ICD‐UX71).^12^, ^14^ Lung sounds were recorded sequentially from eight chest sites and a ninth cheek position (Figure 1). The overall recording duration was approximately 1–2 min. Study staff then deidentified the recordings and uploaded them onto dedicated servers. Johns Hopkins University sound engineers filtered environmental sound contaminations from recordings using an innovative automated multiband denoising filter.^12^, ^14^

Lung sounds were classified according to a previously described methodology.^14^ In brief, each lung sound was randomly assigned to two members of an expert listening panel of eight pediatricians and pediatric‐experienced physicians who were standardized to interpret lung sounds according to a reference panel of previously recorded lung sounds.^14^ After adjudicating interpretation discrepancies, the listening panel assigned each PERCH case 1 prespecified summary lung sound classification.^14^ All summary lung sound classifications were then relabeled post hoc into dichotomous categories according to the hierarchy shown in Table 1. Dichotomous categories positive for abnormal lung sounds, for example, crackles or wheeze, were used as the index test for WHO‐defined primary endpoint pneumonia (radiographic pneumonia) on chest radiography. Members of the chest radiograph reading panel and the lung sound listening panel were masked to the clinical information of study subjects, including lung recording and chest radiograph results.

Institutional review boards responsible for each study site and the Johns Hopkins Bloomberg School of Public Health approved this study.

### 2.4 Statistical analysis

To evaluate associations between lung recordings and radiographic pneumonia or death, we used the *t* test for continuous variables and the Pearson *χ* ^2^ or Fisher exact tests for categorical variables. We calculated unadjusted odds ratios (OR) and 95% confidence intervals (CIs) for radiographic pneumonia (vs. normal) and mortality (vs. alive), as predicted by each lung sound model (abnormal vs. normal) using simple logistic regression. Children with missing or uninterpretable lung sound recordings, or with missing or uninterpretable chest radiographs or radiographs classified as “other infiltrate” only were excluded from analyses comparing lung sounds and chest radiographs. Multiple logistic regression was used to adjust for sex, age, and study site in multivariate analyses. We also conducted a sensitivity analysis using a random‐effects regression model to evaluate the association between lung sounds and radiographic pneumonia using country as the group variable. All statistical analyses were performed using SAS (version 9.4).

## 3 RESULTS

**Figure 2:**
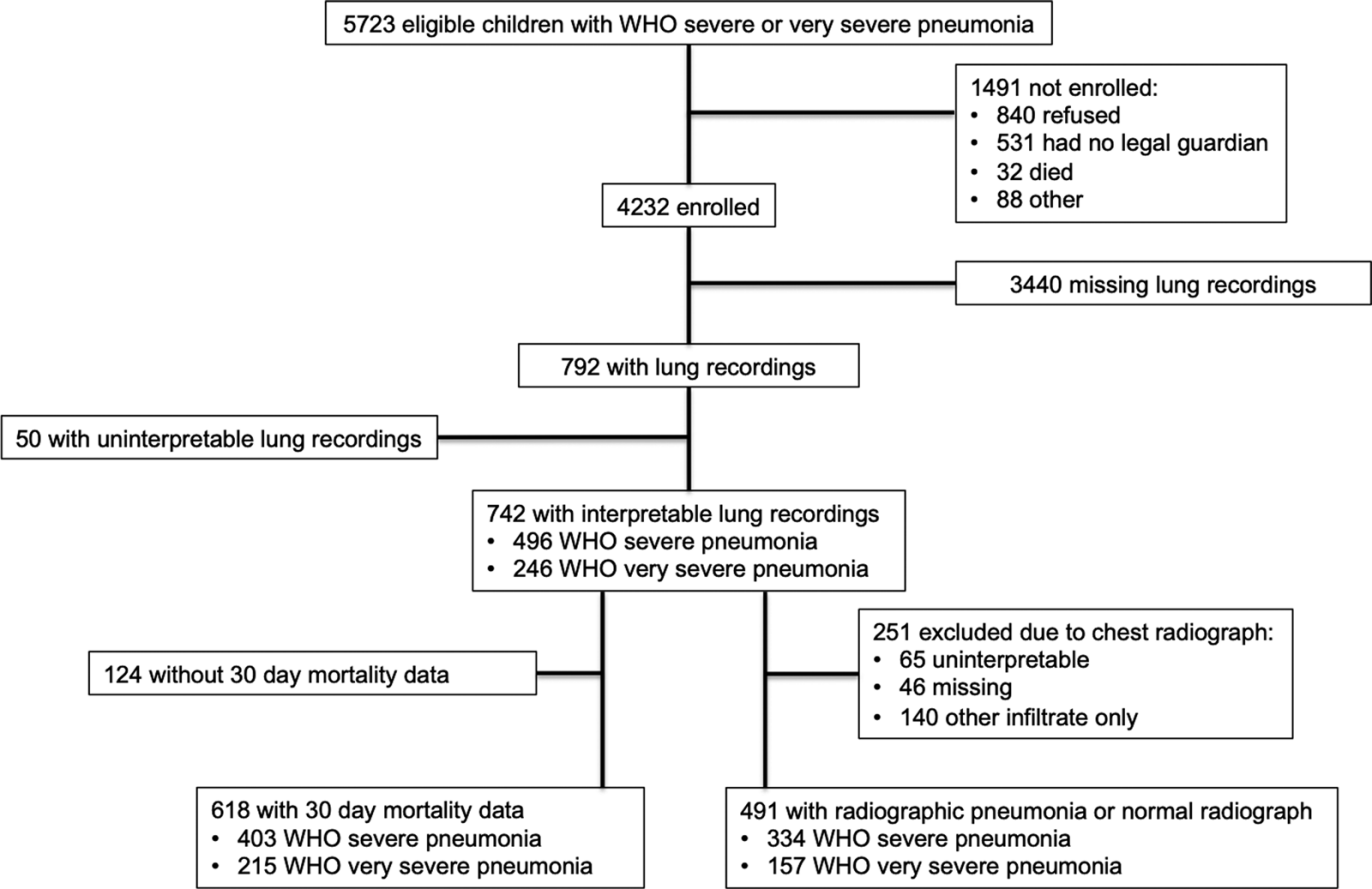
Study flow

Among 792 total PERCH cases with lung sound recordings, 742 children had interpretable recordings (93.6%), 618 (78.0%) had both an interpretable recording and a 30‐day outcome, and 491 children (62.0%) had an interpretable chest radiograph classified as radiographic pneumonia or normal while also having an interpretable lung recording (Figure 2). The median time between acquisition of the digital auscultation recording and chest radiograph was 3.4 h (interquartile range: 0.1–22.0 h).

We described the characteristics of digital auscultation cases in Table 2, and in Table 3 and E‐Table S1 we report participant characteristics according to lung recording and chest radiograph classifications. Among the 742 children with interpretable recordings, 282 (38.0%) were classified as normal, 90 (12.1%) with crackles only, and 370 (49.8%) with wheezing (with or without crackles) (Table 2). Most of the 742 children with interpretable recordings were below one year of age (*n* = 489, 65.9%) and were from African PERCH sites (*n* = 533, 71.8%). Among the 58 children 1–11 months old with an interpretable chest radiograph and crackles only, 48.3% (*n* = 28) had radiographic pneumonia while 32.8% (*n* = 19) had a normal chest radiograph (*p*=.089) (Table 3). By contrast, among the 196 children 1–11 months old with wheezing, 57.1% (*n* = 112) had a normal chest radiograph and 20.9% (*n *= 41) had radiographic pneumonia (*p* < .01). The distribution of lung sound classifications by chest radiograph reading varied substantially across PERCH sites (Table 3).

**Table 2:** Characteristics of digital auscultation cases

			Any wheeze	
Characteristic	Normal lung sound cases, n (%) *N* = 282	Crackle only cases, n (%) *N* = 90	Wheeze only cases, n (%) *N* = 170	Wheeze and crackle cases, n (%) *N* = 200
Age				
1–11 Months	195 (69.1)	68 (75.6)	108 (63.5)	118 (59.0)
12–59 Months	87 (30.9)	22 (24.4)	62 (36.5)	82 (41.0)
Sex				
Female	132 (46.8)	44 (48.9)	60 (35.3)	81 (40.5)
PERCH site				
The Gambia	8 (2.8)	6 (6.7)	40 (23.5)	26 (13.0)
South Africa	38 (13.5)	15 (16.7)	12 (7.1)	29 (14.5)
Zambia	117 (41.5)	34 (37.8)	40 (23.5)	43 (21.5)
Kenya	62 (22.0)	14 (15.6)	27 (15.9)	22 (11.0)
Thailand	23 (8.2)	8 (8.9)	21 (12.4)	12 (6.0)
Bangladesh	34 (12.1)	13 (14.4)	30 (17.6)	68 (34.0)
Clinical				
Premature	32 (11.4)	9 (10.0)	12 (7.1)	13 (6.5)
Never breastfed	20 (7.1)	8 (8.9)	7 (4.1)	13 (6.5)
Illness duration >8 days	22 (7.8)	10 (11.1)	11 (6.5)	10 (5.0)
3 Doses PCV	55 (30.2)	8 (15.1)	35 (29.9)	38 (34.9)
HIV‐infected or ‐exposed	65 (23.0)	18 (20.0)	18 (10.6)	24 (12.0)
Malnutrition (weight‐for‐age)^a^	48 (17.1)	20 (22.5)	18 (10.6)	21 (10.6)
Very severe pneumonia^b^	121 (42.9)	30 (33.3)	46 (27.1)	49 (24.5)
Fast breathing for age^c^	206 (74.4)	79 (90.8)	134 (79.8)	175 (87.9)
Lower chest wall indrawing	231 (81.9)	82 (91.1)	162 (95.3)	194 (97.0)
Hypoxemia^d^	104 (37.1)	39 (43.3)	39 (23.1)	65 (32.5)
Malaria parasitemia^e^	9 (4.8)	4 (7.4)	1 (1.0)	2 (2.3)
Anemia^f^	190 (70.1)	61 (71.8)	102 (67.1)	132 (74.6)
Chest radiograph^g^				
Radiographic pneumonia^h^	65 (25.2)	34 (41.0)	24 (14.6)	46 (24.0)
Other infiltrate only	46 (17.8)	16 (19.3)	35 (21.3)	43 (22.4)
Normal	114 (44.2)	26 (31.3)	89 (54.3)	94 (49.0)
Uninterpretable	33 (12.8)	7 (8.4)	16 (9.8)	9 (4.7)
Outcome^i^				
Death	37 (15.5)	11 (16.2)	11 (7.5)	11 (6.0)

Abbreviations: HIV, human immunodeficiency virus; PCV, pneumococcal conjugate vaccine.

^a^ <−3 *Z* score weight‐for‐age.

^b^ Cough and/or difficult breathing with at least one danger sign.

^c^ Respiratory rate >60 breaths/min <2 months of age, >50 breaths/min 2–<12 months of age, >40 breaths/min >12 months of age.

^d^ Room air oxygen saturation <90% in South Africa and Zambia (high altitude sites), <92% at all other sites, or on supplemental oxygen if a room air oxygen saturation reading was not available.

^e^ Malaria testing was done in Kenya, Gambia, and Zambia.

^f^ Hemoglobin <7.5 g/dl.

^g^ Forty‐five children with interpretable lung recordings were missing chest radiograph results.

^h^ World Health Organization‐defined radiographic primary endpoint pneumonia with or without other infiltrate.

^i^ Death during hospitalization or <30 days after hospital discharge. A total of 107 children with interpretable lung recordings were missing an outcome.

**Table 3:** Chest radiograph findings and digital auscultation sound of interest

		Lung sound classification			
Characteristic^a^	Chest radiographic result	Normal	Crackle only	Any wheeze	Wheeze only
Age					
<12 Months	Pneumonia^b^, n/N (%)	51/147 (34.7)	28/58 (48.3)	41/196 (20.9)	16/92 (17.4)
	Other infiltrate only^c^, n/N (%)	32/147 (21.8)	11/58 (19.0)	43/196 (21.9)	24/92 (26.1)
	Normal^d^, n/N (%)	64/147 (43.5)	19/58 (32.8)	112/196 (57.1)	52/92 (56.5)
>12 Months	Pneumonia^b^, n/N (%)	14/78 (17.9)	6/18 (33.3)	29/135 (21.5)	8/56 (14.3)
	Other infiltrate only^c^, n/N (%)	14/78 (17.9)	5/18 (27.8)	35/135 (25.9)	11/56 (19.6)
	Normal^d^, n/N (%)	50/78 (64.1)	7/18 (38.9)	71/135 (52.6)	37/56 (66.1)
Sex					
Female	Pneumonia^b^, n/N (%)	32/98 (32.7)	13/37 (35.1)	27/128 (21.1)	9/52 (17.3)
	Other infiltrate only^c^, n/N (%)	23/98 (23.5)	10/37 (27.0)	35/128 (27.3)	13/52 (25.0)
	Normal^d^, n/N (%)	43/98 (43.9)	14/37 (37.8)	66/128 (51.6)	30/52 (57.7)
Male	Pneumonia^b^, n/N (%)	33/127 (26.0)	21/39 (53.8)	43/203 (21.2)	15/96 (15.6)
	Other infiltrate only^c^, n/N (%)	23/127 (18.1)	6/39 (15.4)	43/203 (21.2)	22/96 (22.9)
	Normal^d^, n/N (%)	71/127 (55.9)	12/39 (30.8)	117/203 (57.6)	59/96 (61.5)
PERCH site					
The Gambia	Pneumonia^b^, n/N (%)	1/6 (16.7)	2/6 (33.3)	8/60 (13.3)	4/36 (11.1)
	Other infiltrate only^c^, n/N (%)	2/6 (33.3)	3/6 (50.0)	15/60 (25.0)	9/36 (25.0)
	Normal^d^, n/N (%)	3/6 (50.0)	1/6 (16.7)	37/60 (61.7)	23/36 (63.9)
South Africa	Pneumonia^b^, n/N (%)	16/32 (50.0)	6/13 (46.2)	12/39 (30.8)	3/11 (27.3)
	Other infiltrate only^c^, n/N (%)	6/32 (18.8)	5/13 (38.5)	13/39 (33.3)	3/11 (27.3)
	Normal^d^, n/N (%)	10/32 (31.3)	2/13 (15.4)	14/39 (35.9)	5/11 (45.5)
Zambia	Pneumonia^b^, n/N (%)	35/84 (41.7)	15/26 (57.7)	25/64 (39.1)	7/30 (23.3)
	Other infiltrate only^c^, n/N (%)	16/84 (19.0)	3/26 (11.5)	11/64 (17.2)	6/30 (20.0)
	Normal^d^, n/N (%)	33/84 (39.3)	8/26 (30.8)	28/64 (43.8)	17/30 (56.7)
Kenya	Pneumonia^b^, n/N (%)	7/53 (13.2)	5/12 (41.7)	14/44 (31.8)	6/23 (26.1)
	Other infiltrate only^c^, n/N (%)	7/53 (13.2)	2/12 (16.7)	17/44 (38.6)	10/23 (43.5)
	Normal^d^, n/N (%)	39/53 (73.6)	5/12 (41.7)	13/44 (29.5)	7/23 (30.4)
Thailand	Pneumonia^b^, n/N (%)	3/16 (18.8)	3/7 (42.9)	6/31 (19.4)	4/19 (21.1)
	Other infiltrate only^c^, n/N (%)	6/16 (37.5)	1/7 (14.3)	4/31 (12.9)	2/19 (10.5)
	Normal^d^, n/N (%)	7/16 (43.8)	3/7 (42.9)	21/31 (67.7)	13/19 (68.4)
Bangladesh	Pneumonia^b^, n/N (%)	3/34 (8.8)	3/12 (25.0)	5/93 (5.4)	0/29 (0)
	Other infiltrate only^c^, n/N (%)	9/34 (26.5)	2/12 (16.7)	18/93 (19.4)	5/29 (17.2)
	Normal^d^, n/N (%)	22/34 (64.7)	7/12 (58.3)	70/93 (75.3)	24/29 (82.8)
Clinical					
Severe pneumonia^e^	Pneumonia^b^, n/N (%)	39/132 (29.5)	20/51 (39.2)	40/253 (15.8)	13/115 (11.3)
	Other infiltrate only^c^, n/N (%)	31/132 (23.5)	14/51 (27.5)	57/253 (22.5)	28/115 (24.3)
	Normal^d^, n/N (%)	62/132 (47.0)	17/51 (33.3)	156/253 (61.7)	74/115 (64.3)
Very severe pneumonia^f^	Pneumonia^b^, n/N (%)	26/93 (28.0)	14/25 (56.0)	30/78 (38.5)	11/33 (33.3)
	Other infiltrate only^c^, n/N (%)	15/93 (16.1)	2/25 (8.0)	21/78 (26.9)	7/33 (21.2)
	Normal^d^, n/N (%)	52/93 (55.9)	9/25 (36.0)	27/78 (34.6)	15/33 (45.5)
Fast breathing for age^g^	Pneumonia^b^, n/N (%)	64/222 (28.8)	32/73 (44.0)	70/328 (21.3)	24/146 (16.4)
	Other infiltrate only^c^, n/N (%)	45/222 (20.3)	16/73 (21.9)	76/328 (23.2)	34/146 (23.3)
	Normal^d^, n/N (%)	113/222 (50.9)	25/73 (34.2)	182/328 (55.5)	88/146 (60.3)
Lower chest wall indrawing	Pneumonia^b^, n/N (%)	65/225 (28.9)	34/76 (44.7)	70/331 (21.1)	24/148 (16.2)
	Other infiltrate only^c^, n/N (%)	46/225 (20.4)	16/76 (21.1)	78/331 (23.6)	35/148 (23.6)
	Normal^d^, n/N (%)	114/225 (50.7)	26/76 (34.2)	183/331 (55.3)	89/148 (60.1)
Hypoxemia^h^	Pneumonia^b^, n/N (%)	64/223 (28.7)	34/76 (44.7)	70/330 (21.2)	24/147 (16.3)
	Other infiltrate only^c^, n/N (%)	46/223 (20.6)	16/76 (21.1)	77/330 (23.3)	34/147 (23.1)
	Normal^d^, n/N (%)	113/223 (50.7)	26/76 (34.2)	183/330 (55.5)	89/147 (60.5)
HIV‐infected or ‐exposed	Pneumonia^b^, n/N (%)	65/225 (28.9)	34/76 (44.7)	70/331 (21.1)	24/148 (16.2)
	Other infiltrate only^c^, n/N (%)	46/225 (20.4)	16/76 (21.1)	78/331 (23.6)	35/148 (23.6)
	Normal^d^, n/N (%)	114/225 (50.7)	26/76 (34.2)	183/331 (55.3)	89/148 (60.1)
Malnutrition (weight‐for‐age)^i^	Pneumonia^b^, n/N (%)	65/224 (29.0)	33/75 (44.0)	70/330 (21.2)	24/148 (16.2)
	Other infiltrate only^c^, n/N (%)	45/224 (20.1)	16/75 (21.3)	78/330 (23.6)	35/148 (23.6)
	Normal^d^, n/N (%)	114/224 (50.9)	26/75 (34.7)	182/330 (55.2)	89/148 (60.1)
Malaria parasitemia^j^	Pneumonia^b^, n/N (%)	43/143 (30.1)	22/44 (50.0)	47/158 (29.7)	17/83 (20.5)
	Other infiltrate only^c^, n/N (%)	25/143 (17.5)	8/44 (18.2)	39/158 (24.7)	22/83 (26.5)
	Normal^d^, n/N (%)	75/143 (52.4)	14/44 (31.8)	72/158 (45.6)	44/83 (53.0)
Anemia^k^	Pneumonia^b^, n/N (%)	65/217 (30.0)	33/73 (45.2)	67/295 (22.7)	22/133 (16.5)
	Other infiltrate only^c^, n/N (%)	43/217 (19.8)	15/73 (20.5)	68/295 (23.1)	29/133 (21.8)
	Normal^d^, n/N (%)	109/217 (50.2)	25/73 (34.2)	160/295 (54.2)	82/133 (61.7)

Abbreviations: HIV, human immunodeficiency virus; PERCH, Pneumonia Etiology Research For Child Health.

^a^ Numerators are the number of children with the chest radiograph finding and denominators are the number of children with the lung sound classification of interest by characteristic.

^b^ World Health Organization‐defined radiographic primary endpoint pneumonia with or without other infiltrate.

^c^ World Health Organization‐defined other infiltrate only.

^d^ Chest radiograph without radiographic pneumonia and without other infiltrate.

^e^ Cough and/or difficult breathing with lower chest indrawing and no danger signs

^f^ Cough and/or difficult breathing with at least one danger sign.

^g^ Respiratory rate >60 breaths/min <2 months of age, >50 breaths/min 2 to <12 months of age, >40 breaths/min >12 months of age.

^h^ Room air oxygen saturation <90% in South Africa and Zambia (high altitude sites), <92% at all other sites, or on supplemental oxygen if a room air oxygen saturation.

^i^ <−3 *Z* score weight‐for‐age.

^j^ Malaria testing was done in Kenya, Gambia, and Zambia.

^k^ Hemoglobin <7.5 g/dl.

### 3.1 Digitally recorded lung sounds and WHO‐defined radiographic pneumonia

In Table 4 we report on the associations between lung sound recordings and radiographic pneumonia when using normal chest radiographs as the referent. We found a lower adjusted OR (aOR) for radiographic pneumonia (aOR: 0.35; 95% CI: 0.15, 0.82) among children with WHO severe pneumonia and wheezing without crackles, relative to normal lung sounds. By contrast, among children with very severe pneumonia, wheezing with or without crackles (aOR: 2.08; 95% CI: 0.97, 4.45), or crackles only (aOR: 2.75; 95% CI: 0.87, 8.65), were associated with higher odds of radiographic pneumonia, relative to normal lung sounds. However, neither of these associations reached statistical significance. The random effects sensitivity analysis in E‐Table S2 suggests the fixed effect model results in Table 4 are robust to other conditions. In E‐Table S3 we explored the performance of combinations of lung sounds in identifying radiographic pneumonia.

**Table 4:** Association between digitally recorded lung sounds and radiographic pneumonia^a^

WHO clinical pneumonia severity, *N* = 491^b^	Lung sounds^c^	Radiographic pneumonia, n/N (%)^a^	OR (95% CI)	*p* Value	aOR (95% CI)^d^	*p* Value
Severe (*N* = 334)^e^, ^b^	Reference	39/101 (38.6%)	1.00			
	Crackle only (no wheeze)	20/37 (54.1%)	1.87 (0.87, 4.00)	.10	2.13 (0.91, 4.96)	.07
	Wheeze only (no crackle)	13/87 (14.9%)	0.28 (0.13, 0.56)	**<.01**	0.35 (0.15, 0.82)	**.01**
	Any wheeze (with or without crackle)	40/196 (20.4%)	0.41 (0.23, 0.69)	**<.01**	0.63 (0.34, 1.15)	.13
Very severe (*N* = 157)^f^	Reference	26/78 (33.3%)	1.00			
	Crackle only (no wheeze)	14/22 (63.6%)	3.50 (1.30, 9.40)	**.01**	2.75 (0.87, 8.65)	.08
	Wheeze only (no crackle)	11/26 (42.3%)	1.47 (0.59, 3.64)	.40	1.45 (0.53, 3.93)	.46
	Any wheeze (with or without crackle)	30/57 (52.6%)	2.22 (1.10, 4.48)	**.02**	2.08 (0.97, 4.45)	.05

*Note*: Bolded values are statistically significant.

Abbreviations: aOR, adjusted odds ratio; CI, confidence interval; OR, odds ratio; PERCH, Pneumonia Etiology Research for Child Health; WHO, World Health Organization.

^a^ WHO‐defined primary endpoint pneumonia with or without other infiltrate.

^b^ Total cases with interpretable digitally recorded lung sounds and interpretable chest radiograph data with radiographic pneumonia or normal classifications.

^c^ Reference of normal digitally recorded lung sounds.

^d^ Model adjusted for sex, age in months, and PERCH site.

^e^ Cough and/or difficult breathing with lower chest indrawing and no danger signs

^f^ Cough and/or difficult breathing with at least one danger sign.

### 3.2 Digitally recorded lung sounds and mortality

We also examined the association between digitally recorded lung sounds and 30‐day mortality among PERCH cases by logistic regression (Table 5). We found that a model of wheezing, regardless of whether crackles were heard or not, compared to normal lung sounds, was associated with a lower odds of mortality (OR 0.37, *p* = .02) in children with severe WHO‐defined pneumonia. After controlling for the demographic characteristics of sex, age, and study site the model was no longer statistically significant.

**Table 5:** Association between digitally recorded lung sounds and mortality^a^

WHO pneumonia severity (*N* = 618)^b^	Lung sounds^c^	Mortality, n/N (%)^a^	OR (95% CI)	* p* Value	aOR^d^ (95% CI)	*p* Value
Severe (*N* = 403)^e^	Reference	12/125 (9.6%)	1.00			
	Crackle only (no wheeze)	3/40 (7.5%)	0.76 (0.20, 2.85)	.68	1.79 (0.48, 6.63)	.38
	Any crackle (with or without wheeze)	8/177 (4.5%)	0.45 (0.17, 1.12)	.08	1.19 (0.45, 3.10)	.72
	Wheeze only (no crackle)	4/101 (4.0%)	0.39 (0.12, 1.24)	.10	1.01 (0.30, 3.42)	.98
	Any wheeze (with or without crackle)	9/238 (3.8%)	0.37 (0.15, 0.90)	**.02**	1.02 (0.39, 2.61)	.97
Very severe (*N* = 215)^f^	Reference	24/110 (21.8%)	1.00			
	Crackle only (no wheeze)	8/25 (32.0%)	1.69 (0.64, 4.37)	.27	1.51 (0.50, 4.57)	.46
	Any crackle (with or without wheeze)	14/64 (21.9%)	1.00 (0.47, 2.11)	.99	1.05 (0.45, 2.41)	.91
	Wheeze only (no crackle)	7/41 (17.1%)	0.74 (0.29, 1.87)	.52	0.93 (0.34, 2.53)	.88
	Any wheeze (with or without crackle)	13/80 (16.3%)	0.70 (0.32, 1.46)	.33	0.75 (0.34, 1.67)	.48

*Note*: Bolded values are statistically significant.

Abbreviations: aOR, adjusted odds ratio; CI, confidence interval; OR, odds ratio; PERCH, Pneumonia Etiology Research for Child Health; WHO, World Health Organization.

^a^ Death during hospitalization or <30 days after hospital discharge.

^b^ Total cases with interpretable digital lung recordings and mortality data.

^c^ Reference of normal digitally recorded lung sounds.

^d^ Model adjusted for gender, age in months, and PERCH site.

^e^ Cough and/or difficult breathing with lower chest indrawing and no danger signs.

^f^ Cough and/or difficult breathing with at least one danger sign.

We additionally examined 30‐day mortality by stratifying for pneumonia severity and digitally recorded lung sound results (Table 6) as well as for selected mortality risk factors (E‐Table 4). Wheezing, regardless of crackles, was associated with lower mortality among children with severe pneumonia (3.8% [9/238] vs. 9.1% [15/165], *p* = .03) (Table 6), children 1–11 months (7.3% [14/191] vs. 20.0% [40/200], *p* < .01) (E‐Table S4), children without hypoxemia (3.0% [7/232] vs. 9.0% [17/189], *p* = .01) and children with anemia (9.1% [18/197] vs. 19.1% [38/199], *p* < .01) (E‐Table S4). Among children without severe malnutrition, wheezing (regardless of crackles) was associated with lower mortality (6.5%, [19/299] vs. 13.9% [33/238], *p* < .01), while crackles only were associated with higher mortality (18.9% [10/53] vs. 8.8% [42/477], *p* = .02) (E‐Table S4). In E‐Table S5 we described case fatality for combinations of lung sounds and chest radiograph findings.

**Table 6:** Case fatality ratio^a^ stratified by digitally recorded lung sounds and WHO pneumonia severity

*N* = 618^b^		Crackle only (no wheeze)			Any crackle (with or without wheeze)			Wheeze only (no crackle)			Any wheeze (with or without crackle)		
		Yes *N* = 65	No *N* = 553	*p* Value	Yes *N* = 241	No *N* = 377	*p* Value	Yes *N* = 142	No *N* = 476	*p* Value	Yes *N* = 318	No *N* = 300	*p* Value
Deaths and WHO pneumonia severity, n/N (%)	All	11/65 (16.9%)	58/553 (10.5%)	.14	22/241 (9.1%)	47/377 (12.5%)	.23	11/142 (7.7%)	58/476 (12.2%)	.17	22/318 (6.9%)	47/300 (15.7%)	**<.01**
	Severe^c^	3/40 (7.5%)	21/363 (5.8%)	.72	8/177 (4.5%)	16/226 (7.1%)	.29	4/101 (4.0%)	20/302 (6.6%)	.46	9/238 (3.8%)	15/165 (9.1%)	**.03**
	Very severe^d^	8/25 (32.0%)	37/190 (19.5%)	.18	14/64 (21.9%)	31/151 (20.5%)	.85	7/41 (17.1%)	38/174 (21.8%)	.67	13/80 (16.3%)	32/135 (23.7%)	.22

*Note*: Bolded values are statistically significant.

Abbreviations: HIV, human immunodeficiency virus; PERCH, Pneumonia Etiology Research for Child Health; WHO, World Health Organization.

^a^ Death during hospitalization or <30 days after hospital discharge

^b^ Total cases with interpretable digital lung recordings and mortality data

^c^ Cough and/or difficult breathing with lower chest indrawing and no danger signs

^d^ Cough and/or difficult breathing with at least one danger sign.

## 4 DISCUSSION

This study examined the association of digitally recorded lung sounds with WHO‐defined radiographic primary endpoint pneumonia and mortality among children 1–59 months old hospitalized with pneumonia from eight sites in six African and Asian countries participating in the PERCH Study. Using PERCH data we previously reported that our recording techniques, ambient sound filtering, and interpretation methods were likely valid, achieving >90% interpretability, moderate between‐listener agreement, and a high proportion of normal lung sound recordings among controls, compared to clinical pneumonia cases.^14^ We have also developed and internally validated a fully automated lung sound processing algorithm that can identify abnormal lung sounds from PERCH recordings with nearly 90% accuracy.^13^ In this research, we extend this initial body of work to show that human interpretation of digital lung recordings has important clinical relationships with radiographic pneumonia and pneumonia mortality. While these results are encouraging it is important to stress that they should be considered as only an initial step towards clinical or research application given the lack of a gold standard for pneumonia diagnosis and the inherent limitations of the WHO‐defined radiographic pneumonia methodology, as discussed below. Additional research evaluating digital auscultation as a potential diagnostic tool for pediatric respiratory illnesses will be required before considering it for clinical implementation.

Although chest radiographs are considered the reference standard for pneumonia diagnosis among children, radiographic imaging exposes children to ionizing radiation.^19^ Furthermore, radiographic equipment is expensive, facility‐based, and there is a lack of interpretation expertise in most LMICs.^20^ All of these issues pose barriers to wide‐scale implementation of chest radiography in LMICs. Digital stethoscopes that incorporate an automated lung sound processing algorithm, on the other hand, circumvent these obstacles and have the potential to be a community‐based, noninvasive point‐of‐care pneumonia diagnostic. Understanding the relationships between lung recordings and radiographic pneumonia in LMICs is, therefore, crucial, but has yet to be rigorously studied.

After controlling for demographic characteristics we found that wheezing among children with WHO severe pneumonia (i.e., no WHO danger signs) is independently associated with a lower odds of radiographic pneumonia (OR: 0.38; 95% CI: 0.15, 0.92). In contrast, we also found a higher odds of radiographic pneumonia in children with lung recordings of crackles or wheezes and WHO very severe pneumonia (i.e., with danger signs), although these results did not reach statistical significance (95% CIs crossed 1.0). We may have observed qualitatively conflicting odds of radiographic pneumonia by pneumonia severity strata because children with more severe illness (i.e., WHO very severe disease) and wheezing may have more severely narrowed airways and greater airflow obstruction, both of which could subsequently result in hyperinflation and atelectasis (i.e., collapsed areas of lung parenchyma) on chest radiographs that could be secondarily infected with bacteria.^21^ A higher proportion of these more severely ill children may also have had primary bacterial pneumonia with alveolar consolidation present on imaging. Importantly, the WHO radiographic primary endpoint pneumonia does not differentiate between alveolar consolidation and atelectasis.^16^, ^20^ Overall the results from this analysis indicate that lung recordings have potential for use as a pneumonia diagnostic among children.

We previously found wheezing to be the most common abnormal recorded lung sound heard among PERCH cases, identified in about 50% of children with WHO severe or very severe pneumonia.^14^ This study now suggests that wheezes heard on lung recordings are not only common but may be associated with a lower risk of mortality among children with certain characteristics. Specifically, wheezing children, with or without crackles, who also had either chest indrawing (i.e., WHO severe pneumonia), or had no severe malnutrition or no hypoxemia had lower case fatality compared to children without these characteristics. We also found that wheezing, with or without crackles, was associated with a lower odds of mortality among children with WHO severe pneumonia using simple logistic regression. However, this association lacked significance after adjustment, suggesting that other risk factors likely confound the wheezing‐mortality relationship. This notion is further supported by our observation that among sicker children with WHO very severe pneumonia (i.e., with danger signs), lung sound recordings with crackles or wheeze also had no statistically significant associations with mortality. As mentioned previously, we also may have found wheezing to lack association with lower mortality among more severely ill children since more severe airway narrowing itself can lead to airflow obstruction, hyperinflation, atelectasis, ventilation‐perfusion mismatch, and ultimately respiratory failure and death.^21^ Other studies in LMICs have published findings that children with WHO pneumonia and wheeze without danger signs also have lower mortality.^22^, ^23^ These studies lend further validity to our work given their observations of the wheeze‐mortality relationship were based on real‐time interpretation of lung sounds from traditional acoustic stethoscopes.

Among the nine children with wheezing and WHO severe pneumonia who died in this study (Table 6), all had an additional risk factor for mortality that was identifiable at enrollment, suggesting that these children could be flagged as high‐risk and treated accordingly. Taken together these findings imply that lower‐risk wheezing children identified by digital auscultation could potentially be treated according to a different management algorithm than those without wheezing or risk factors for adverse outcomes. Given the emerging global crisis of antimicrobial resistance, strategies that safely reduce unnecessary antibiotic exposure are urgently required.^7^ Further research investigating whether digital auscultation may be an effective modality for achieving rational antibiotic use among carefully selected children with low‐risk WHO pneumonia is needed.

This study has three important limitations. First, due to human resource constraints, participants were not sampled consecutively at three of the eight study sites. To evaluate whether selection bias may have affected our results, we compared PERCH digital auscultation participants to nonparticipants. We found that digital auscultation participants were less severely ill than nonparticipants since a lower proportion of participants, compared to nonparticipants, were based at the African study sites, had severe malnutrition, and had hypoxemia (E‐Table S1). As a result, if any bias exists, we believe our results are biased toward more conservative inferences. The second main limitation to this study is that pneumonia has no true gold standard reference.^24^ Although chest radiographs are considered the best current reference standard, they are not ideal given the interpretation of radiographic abnormalities is subjective and the appearance of radiographic abnormalities can also lag behind clinical signs, potentially reducing sensitivity and delaying effective treatment. It is well known that normal chest radiographs can be present in children with signs consistent with clinical pneumonia, and this also occurred in PERCH, as 46% of children meeting WHO clinical pneumonia criteria had normal chest radiographs.^25^ It is also important to note that the WHO radiographic primary endpoint pneumonia definition is not intended for clinical application, and this limits the clinical generalizability of these results. However, despite chest radiographs serving as the reference standard for pneumonia diagnosis there are few chest radiograph interpretation schemas used as widely as the WHO method. Although this interpretation approach was initially established for the evaluation of bacterial conjugate vaccine efficacy its application has been extended to epidemiologic research of child pneumonia in LMICs,^16^ including as the reference standard in the PERCH Study.^5^ It is important to note that not all experts agree with application of the WHO chest radiograph methodology for epidemiologic research due to its bias toward specificity rather than sensitivity, leading to underestimation of the public health burden of pneumonia. These results should be interpreted within this context. Despite the inherent limitations to chest radiographs in general and the WHO method itself, PERCH applied rigorous interpretation procedures to optimize interpretation reliability and case ascertainment, achieving 78% agreement between primary readers of radiographs (Cohen's kappa: 0.50), which was comparable to other studies using this WHO methodology.^15^, ^26^ Lastly, all children enrolled into PERCH met clinical pneumonia criteria, which does not allow us to assess digital auscultation utility among children without clinical pneumonia. Such an evaluation is an important next step.

In summary, the results of this study suggest that digital lung recordings may have a future role in pediatric respiratory research and as a point‐of‐care respiratory diagnostic and prognosticating tool for children in LMICs. Essential next steps include evaluating the feasibility and decision‐making impact of digital stethoscope use by both formal and informally trained health workers, evaluating it against other pneumonia reference endpoints other than chest radiography, assessing agreement between standard auscultation by experts with digital recorded lung sounds interpreted by either humans or automated algorithms, and externally validating the automated lung sound processing algorithm in other similarly vulnerable pediatric populations.

## CONFLICT OF INTERESTS

The authors declare that there are no conflict of interests.

## Supporting information

Supporting information.

Supporting information.

Supporting information.

Supporting information.

Supporting information.
