# Supplementary material for: Digital auscultation in PERCH: Associations with chest radiography and pneumonia mortality in children
Source: Pediatr Pulmonol. 2020 Sep 11;55(11):3197–208. doi: 10.1002/ppul.25046 (PMC7692889; doi:10.1002/ppul.25046)
Supplement: Supplementary file 1 — Supporting information. [file PPUL-55-3197-s001.docx]

E-table 1. PERCH digital auscultation case participants and non-participants

| Characteristic | Digital auscultation participants  N=792 | Digital auscultation non-participants  N=3439 | P value |
| --- | --- | --- | --- |
| Females, n/N (%) | 343 (43.3%) | 1473 (42.8%) | 0.81 |
| Age, mean (SD) | 11.3 (11.6) | 11.6 (11.5) | 0.45 |
| African region, n/N (%) | 580 (73.2%) | 2902 (84.4%) | <0.01 |
| HIV-infected or –exposed, n/N (%) | 134 (16.9%) | 563 (16.4%) | 0.70 |
| Very severe pneumonia, n/N (%)* | 263 (33.2%) | 1106 (32.2%) | 0.57 |
| Severe malnutrition, n/N (%)†,‡ | 76/766 (9.9%) | 422/3337 (12.6%) | 0.03 |
| Bacteremia, n/N (%)‡ | 24/758 (3.2%) | 146/3417 (4.3%) | 0.16 |
| Malaria parasitemia, n/N (%)‡ | 17/464 (3.7%) | 75/2040 (3.7%) | 0.98 |
| Hypoxemia, n/N (%)§ | 267/789 (33.8%) | 1321/3432 (38.5%) | 0.01 |
| Hospital mortality, n/N (%)‡ | 61/790 (7.7%) | 256/3433 (7.5%) | 0.79 |
| Radiographic pneumonia, n/N (%)‡,ll | 102/746 (13.7%) | 444/3226 (13.8%) | 0.94 |

PERCH indicates Pneumonia Etiology Research for Child Health; SD, standard deviation; HIV, human immunodeficiency virus; WHO, World Health Organization.

*Cough and/or difficult breathing with at least one danger sign.

†<-3 z-score weight-for-age

‡N differs from total N due to missing data.

§Room air oxygen saturation <90% in South Africa and Zambia (high altitude sites), <92% at all other sites, or on supplemental oxygen if a room air oxygen saturation reading was not available

llWHO primary endpoint pneumonia with or without other infiltrate
