# Supplementary material for: Digital auscultation in PERCH: Associations with chest radiography and pneumonia mortality in children
Source: Pediatr Pulmonol. 2020 Sep 11;55(11):3197–208. doi: 10.1002/ppul.25046 (PMC7692889; doi:10.1002/ppul.25046)
Supplement: Supplementary file 2 — Supporting information. [file PPUL-55-3197-s002.docx]

E-table 2. Association between digitally recorded lung sounds and radiographic pneumonia* using random effects logistic regression

| WHO clinical pneumonia severity, N=491† | Lung sounds‡ | Radiographic pneumonia*, n/N (%) | OR (95% CI) | p value | aOR (95% CI)§ | p value |
| --- | --- | --- | --- | --- | --- | --- |
| Severell (N=334) | Reference | 39/101 (38.6%) | 1.00 |  |  |  |
|  | Crackle only (no wheeze) | 20/37 (54.1%) | 1.87 (0.87, 4.00) | 0.10 | 2.16 (0.95, 4.95) | 0.07 |
|  | Wheeze only (no crackle) | 13/87 (14.9%) | 0.28 (0.13, 0.56) | **<0.01** | 0.33 (0.15, 0.77) | **0.01** |
|  | Any wheeze (with or without crackle) | 40/196 (20.4%) | 0.41 (0.23, 0.69) | **<0.01** | 0.59 (0.32, 1.08) | 0.09 |
| Very severe†† (N=157) | Reference | 26/78 (33.3%) | 1.00 |  |  |  |
|  | Crackle only (no wheeze) | 14/22 (63.6%) | 3.50 (1.30, 9.40) | **0.01** | 2.90 (0.92, 9.13) | 0.07 |
|  | Wheeze only (no crackle) | 11/26 (42.3%) | 1.47 (0.59, 3.64) | 0.40 | 1.41 (0.53, 3.74) | 0.49 |
|  | Any wheeze (with or without crackle) | 30/57 (52.6%) | 2.22 (1.10, 4.48) | **0.02** | 2.04 (0.97, 4.30) | 0.06 |

WHO indicates World Health Organization; PERCH, Pneumonia Etiology Research for Child Health; OR, odds ratio; CI, confidence interval; aOR, adjusted odds ratio.

*WHO-defined primary endpoint pneumonia with or without other infiltrate

†Total cases with interpretable digitally recorded lung sounds and interpretable chest radiograph data with radiographic pneumonia or normal classifications.

‡Reference of normal digitally recorded lung sounds.

§Model adjusted for sex and age in months as a fixed effect and PERCH site as a random effect.

llCough and/or difficult breathing with lower chest indrawing and no danger signs

††Cough and/or difficult breathing with at least one danger sign.
