# Supplementary material for: Digital auscultation in PERCH: Associations with chest radiography and pneumonia mortality in children
Source: Pediatr Pulmonol. 2020 Sep 11;55(11):3197–208. doi: 10.1002/ppul.25046 (PMC7692889; doi:10.1002/ppul.25046)
Supplement: Supplementary file 3 — Supporting information. [file PPUL-55-3197-s003.docx]

E-table 3. Diagnostic performance of digitally recorded lung sounds for radiographic pneumonia

| WHO pneumonia severity, N=491^2^ | Lung sounds | Sensitivity, n/N (%) (95% CI) | Specificity, n/N (%) (95% CI) | PPV, n/N (%) (95% CI) | NPV, n/N (%) (95% CI) | Likelihood ratio positive, n/N (%) (95% CI) | Likelihood ratio negative, n/N (%) (95% CI) |
| --- | --- | --- | --- | --- | --- | --- | --- |
| Severe (N=334) | Crackle only (no wheeze) | 20/99 (20.2%) (12.8%, 29.5%) | 218/235 (92.8%) (88.7%, 95.7%) | 20/37 (54.1%) (36.9%, 70.5%) | 218/297 (73.4%) (68.0%, 78.3%) | 2.7 (1.5, 5.1) | 0.8 (0.7, 0.9) |
|  | Any crackle (with or without wheeze) | 47/99 (47.5%) (37.3%, 57.8%) | 136/235 (57.9%) (51.3%, 64.3%) | 47/146 (32.2%) (24.7%, 40.4%) | 136/188 (72.3%) (65.4%, 78.6%) | 1.1 (0.8, 1.4) | 0.9 (0.7, 1.1) |
|  | Wheeze only (no crackle) | 13/99 (13.1%) (7.2%, 21.4%) | 161/235 (68.5%) (62.2%, 74.4%) | 13/87 (14.9%) (8.2%, 24.2%) | 161/247 (65.2%) (58.9%, 71.1%) | 0.4 (0.2, 0.7) | 1.2 (1.1, 1.4) |
|  | Any wheeze (with or without crackle) | 40/99 (40.4%) (30.7%, 50.7%) | 79/235 (33.6%) (27.6%, 40.0%) | 40/196 (20.4%) (15.0%, 26.7%) | 79/138 (57.2%) (48.5%, 65.6%) | 0.6 (0.4, 0.7) | 1.7 (1.3, 2.2) |
| Very severe (N=157) | Crackle only (no wheeze) | 14/70 (20.0%) (11.4%, 31.3%) | 79/87 (90.8%) (82.7%, 95.9%) | 14/22 (63.6%) (40.7%, 82.8%) | 79/135 (58.5%) (49.7%, 66.9%) | 2.1 (0.9, 4.8) | 0.8 (0.7, 1.0) |
|  | Any crackle (with or without wheeze) | 33/70 (47.1%) (35.1%, 59.4%) | 67/87 (77.0%) (66.8%, 85.4%) | 33/53 (62.3%) (47.9%, 75.2%) | 67/104 (64.4%) (54.4%, 73.6%) | 2.0 (1.2, 3.2) | 0.6 (0.5, 0.8) |
|  | Wheeze only (no crackle) | 11/70 (15.7%) (8.1%, 26.4%) | 72/87 (82.8%) (73.2%, 90.0%) | 11/26 (42.3%) (23.4%, 63.1%) | 72/131 (55.0%) (46.0%, 63.7%) | 0.9 (0.4, 1.8) | 1.0 (0.8, 1.1) |
|  | Any wheeze (with or without crackle) | 30/70 (42.9%) (31.1%, 55.3%) | 60/87 (69.0%) (58.1%, 78.5%) | 30/57 (52.6%) (39.0%, 66.0%) | 60/100 (60.0%) (49.7%, 69.7%) | 1.3 (0.9, 2.0) | 0.8 (0.6, 1.0) |

WHO indicates World Health Organization; PERCH, Pneumonia Etiology Research for Child Health; CI, confidence interval; PPV, positive predictive value; NPV, negative predictive value.

^1^WHO primary endpoint pneumonia with or without other infiltrate.

^2^Total cases with interpretable digitally recorded lung sounds and interpretable chest radiograph data.
