# Supplementary material for: Digital auscultation in PERCH: Associations with chest radiography and pneumonia mortality in children
Source: Pediatr Pulmonol. 2020 Sep 11;55(11):3197–208. doi: 10.1002/ppul.25046 (PMC7692889; doi:10.1002/ppul.25046)
Supplement: Supplementary file 4 — Supporting information. [file PPUL-55-3197-s004.docx]

E-table 4. Case fatality ratio* stratified by digitally recorded lung sounds and mortality risk factors

| N=618† | | Crackle only (no wheeze) | | | Any crackle (with or without wheeze) | | | Wheeze only (no crackle) | | | Any wheeze (with or without crackle) | | |
| --- | --- | --- | --- | --- | --- | --- | --- | --- | --- | --- | --- | --- | --- |
|  |  | Yes  N=65 | No  N=553 | P value | Yes  N=241 | No  N=377 | P value | Yes  N=142 | No  N=476 | P value | Yes  N=318 | No  N=300 | P value |
| Deaths and Age, in months, n/N (%) | 1-11 | 9/46 (19.6%) | 45/345 (13.0%) | 0.25 | 15/151 (9.9%) | 39/240 (16.3%) | 0.09 | 8/86 (9.3%) | 46/305 (15.1%) | 0.21 | 14/191 (7.3%) | 40/200 (20.0%) | **<0.01** |
|  | 12-23 | 2/6 (33.3%) | 9/125 (7.2%) | 0.08 | 6/52 (11.5%) | 5/79 (6.3%) | 0.34 | 2/34 (5.9%) | 9/97 (9.3%) | 0.72 | 6/80 (7.5%) | 5/51 (9.8%) | 0.75 |
|  | 24-59 | 0/13 (0%) | 4/83 (4.8%) | 1.00 | 1/38 (2.6%) | 3/58 (5.2%) | 1.00 | 1/22 (4.5%) | 3/74 (4.1%) | 1.00 | 2/47 (4.3%) | 2/49 (4.1%) | 1.00 |
| Deaths and HIV status, n/N (%) | HIV-infected | 3/6 (50.0%) | 19/41 (46.3%) | 1.00 | 6/13 (46.2%) | 16/34 (47.1%) | 1.00 | 0/4 (0%) | 22/43 (51.2%) | 0.11 | 3/11 (27.3%) | 19/36 (52.8%) | 0.17 |
|  | HIV-uninfected | 7/54 (13.0%) | 36/475 (7.6%) | 0.18 | 13/216 (6.0%) | 30/313 (9.6%) | 0.14 | 11/121 (9.1%) | 32/408 (7.8%) | 0.70 | 17/283 (6.0%) | 26/246 (10.6%) | 0.07 |
| Deaths and Severe malnutrition, n/N (%)ll | Severe malnutritionll | 1/9 (11.1%) | 9/60 (15.0%) | 1.00 | 1/20 (5.0%) | 9/49 (18.4%) | 0.26 | 2/9 (22.2%) | 8/60 (13.3%) | 0.60 | 2/20 (10.0%) | 8/49 (16.3%) | 0.71 |
|  | No severe malnutrition | 10/53 (18.9%) | 42/477 (8.8%) | **0.02** | 21/213 (9.9%) | 31/317 (9.8%) | 1.00 | 8/132 (6.1%) | 44/398 (11.1%) | 0.12 | 19/292 (6.5%) | 33/238 (13.9%) | **<0.01** |
| Deaths and Hypoxemia, n/N (%)†† | Hypoxemia†† | 7/25 (28.0%) | 38/169 (22.5%) | 0.61 | 15/81 (18.5%) | 30/113 (26.5%) | 0.22 | 7/29 (24.1%) | 38/165 (23.0%) | 1.00 | 15/85 (17.6%) | 30/109 (27.5%) | 0.12 |
|  | No hypoxemia | 4/40 (10.0%) | 20/381 (5.2%) | 0.26 | 7/160 (4.4%) | 17/261 (6.5%) | 0.39 | 4/112 (3.6%) | 20/309 (6.5%) | 0.34 | 7/232 (3.0%) | 17/189 (9.0%) | **0.01** |
| Deaths and Anemia, n/N (%)‡‡ | Anemia‡‡ | 10/46 (21.7%) | 46/350 (13.1%) | 0.11 | 19/158 (12.0%) | 37/238 (15.5%) | 0.37 | 9/85 (10.6%) | 47/311 (15.1%) | 0.38 | 18/197 (9.1%) | 38/199 (19.1%) | **<0.01** |
|  | No anemia | 0/15 (0%) | 10/156 (6.4%) | 0.60 | 2/58 (3.4%) | 8/113 (7.1%) | 0.49 | 2/42 (4.8%) | 8/129 (6.2%) | 1.00 | 4/85 (4.7%) | 6/86 (7.0%) | 0.74 |

PERCH indicates Pneumonia Etiology Research for Child Health; WHO, World Health Organization; HIV, human immunodeficiency virus.

*Death during hospitalization or <30 days after hospital discharge

†Total cases with interpretable digital lung recordings and mortality data

ll<-3 z-score weight-for-age

††Room air oxygen saturation <90% in South Africa and Zambia (high altitude sites), <92% at all other sites, or on supplemental oxygen if a room air oxygen saturation reading was not available

‡‡Hemoglobin <7.5 g/dL
