# Supplementary material for: Digital auscultation in PERCH: Associations with chest radiography and pneumonia mortality in children
Source: Pediatr Pulmonol. 2020 Sep 11;55(11):3197–208. doi: 10.1002/ppul.25046 (PMC7692889; doi:10.1002/ppul.25046)
Supplement: Supplementary file 5 — Supporting information. [file PPUL-55-3197-s005.docx]

E-table 5. Case fatality for combinations of lung sounds and chest radiograph findings

| Lung sound model | Crackle only | | Any wheeze | | Normal | | Crackle only | Any wheeze | Normal |
| --- | --- | --- | --- | --- | --- | --- | --- | --- | --- |
| Chest radiograph | Radiographic pneumonia* | Other infiltrate only | Radiographic pneumonia* | Other infiltrate only | Radiographic pneumonia* | Other infiltrate only | Normal | | |
| Death† | 5/24 (20.8%) | 0/14 (0%) | 8/63 (12.7%) | 3/69 (4.3%) | 10/54 (18.5%) | 2/38 (5.3%) | 3/19 (15.8%) | 7/170 (4.1%) | 4/97 (4.1%) |

*WHO-defined primary endpoint pneumonia with or without other infiltrate

†Death during hospitalization or <30 days after hospital discharge
